# Corona Enhancement and Mosaic Architecture for Prognosis and Selection Between of Liver Resection Versus Transcatheter Arterial Chemoembolization in Single Hepatocellular Carcinomas >5 cm Without Extrahepatic Metastases: An Imaging-Based Retrospective Study

**DOI:** 10.1097/MD.0000000000002458

**Published:** 2016-01-15

**Authors:** Meng Li, Yongjie Xin, Sirui Fu, Zaiyi Liu, Yong Li, Baoshan Hu, Shuting Chen, Changhong Liang, Ligong Lu

**Affiliations:** From the Southern Medical University, Guangzhou, China (ML, SC); Department of Radiology, Guangdong General Hospital, Guangdong Academy of Medical Sciences, Guangzhou, China (ML, ZL, SC, CL); and Department of Interventional Radiology, Guangdong Provincial Cardiovascular Institute, Guangdong General Hospital, Guangdong Academy of Medical Sciences, Guangzhou, China (YX, SF, YL, BH, LL).

## Abstract

Corona enhancement and mosaic architecture are 2 radiologic features of hepatocellular carcinoma (HCC). However, neither their prognostic values nor their impacts on the selection of liver resection (LR) versus transcatheter arterial chemoembolization (TACE) as treatment modalities have been established.

We retrospectively analyzed 275 patients with a single HCC lesion >5 cm without extrahepatic metastasis treated with LR or TACE. In LR patients, the overall survival (OS) and time to progression (TTP) were compared between corona enhancement negative (corona−) versus positive (corona+) and mosaic architecture negative (mosaic−) versus positive (mosaic+) patients. Furthermore, by the combination of corona and mosaic, LR patients were divided into negative for both corona and mosaic patterns (LR−/−), positive for only 1 feature (LR+/−), and positive for both (LR+/+); their OS and TTP were compared to those of the TACE group. Cox regression was performed to identify independent factors for OS.

In the survival plots for LR, corona− had better OS and TTP than corona+, and mosaic− had better OS than mosaic+. There was no significant difference in TTP between the subgroups. On Cox regression analysis, corona enhancement, but not mosaic architecture, was a significant factor for OS, whereas neither were a significant factor for TTP. In TACE patients, neither corona nor mosaic patterns had significant correlations with OS or TTP. In the whole population, LR−/ and LR+/− subgroups had similar OS, which was better than the LR+/+ and TACE groups. Moreover, LR−/− and LR+/− patients had better TTP than TACE patients, but there were no differences between the LR−/− versus LR+/−, LR−/ versus LR+/+, LR+/− versus LR+/+, and LR+/+ versus TACE groups. On Cox regression analysis, the presence of corona/mosaic patterns was an independent prognostic factor for OS.

Our results showed that, for patients with a single HCC >5 cm without extrahepatic metastasis, corona and mosaic patterns are indicators of limited LR efficacy. When both of the features are present, TACE can be used instead of LR with no negative influence on survival.

## INTRODUCTION

Liver cancer is the fifth and seventh most frequently diagnosed cancer, ranking second and sixth in the causes of cancer-related deaths, for men and women, respectively.^1^ Hepatocellular carcinoma (HCC) represents the majority of liver cancer types.^2,3^ With the availability of multiple treatment modalities, including liver resection (LR), transcatheter arterial chemoembolization (TACE), and ablation, choosing an appropriate treatment strategy is a critical decision for oncologists. The Barcelona Clinic Liver Cancer (BCLC) staging system, which links therapeutic strategies to treatment outcomes, is commonly used to decide treatment modalities. BCLC is a well-recognized staging system for HCC and has been adopted by the European Association for the Study of the Liver and the European Organization for Research and Treatment of Cancer, as well as by the American Association for the Study of Liver Diseases.

According to the BCLC staging system, LR is recommended for HCC patients up to stage A disease, while TACE is the best choice for stage B.^4,5^ However, controversies have recently arisen regarding treatment strategies in certain situations, such as single tumors >5 cm without vascular invasion or extrahepatic metastasis,^6–11^ which was defined as stage AB in 1 study.^9^ This study showed that, regardless of BCLC stage, LR resulted in a greater survival benefit than locoregional therapy such as TACE and ablation.^9^ Another study showed that, for HCC patients with resectable multiple lesions with a BCLC stage higher than A, LR resulted in better survival than conventional TACE.^12^ In our clinical practice, however, not all patients with BCLC stages above A exhibited a greater survival benefit with LR. Moreover, LR may increase perioperative complications and prolong postoperative recovery compared to TACE. Therefore, a more reliable method to select the most appropriate treatment modality is desirable.

For years, prognostic studies mainly focused on clinical or biological markers.^13–15^ However, routinely acquired computed tomography (CT) and magnetic resonance (MR) images were largely undervalued. Endorsed by major clinical practice guidelines.^4,5^ CT and MR imaging played emerging crucial roles in the diagnosis, staging, and characterization of HCC. Although the use of hepatobiliary agents for MR imaging can provide additional diagnostic information (especially for small HCC),^16,17^ studies of imaging features as they pertain to HCC prognosis and patient selection are limited.

Corona enhancement and mosaic architecture are 2 imaging features favoring the diagnosis of large HCC.^18^ Corona is pointing to enhancement of the venous drainage area in the peritumoral parenchyma.^19^ Studies shown that corona enhancement might convey information on microvascular invasion and metastatic satellites.^20–22^ HCCs with corona enhancement findings tend to be diagnosed as progressed, hypervascular HCC.^18^

Mosaic architecture is the presence of randomly distributed internal nodules or compartments within a mass that differ in shape on enhancement.^18^ Mosaic patterns on characterization and differential diagnosis were previously described in Yersinia colitis.^23^ In HCC, mosaic architecture is characteristic of tumor heterogeneity with histologic and cytologic variations more common in large HCC,^24^ manifesting by confounding factors such as fibrous separations, necrosis, hemorrhage, copper deposition, and fatty infiltration.^25^

In our previous studies, we found that corona and mosaic patterns were correlated with survival outcomes in HCC patients treated with LR but not TACE (unpublished data). Therefore, we postulate that these 2 imaging features may provide reliable information for HCC prognosis and provide better guidance with respect to treatment strategies. To test our hypothesis, we conducted this study on patients with a single HCC lesion above BCLC stage A (>5 cm) with no extrahepatic metastases.

## METHODS

### Patients

This study was conducted under the approval of the Ethics Committee of Guangdong General Hospital. The requirement for informed consent was waived because of the study's retrospective nature.

**FIGURE 1:**
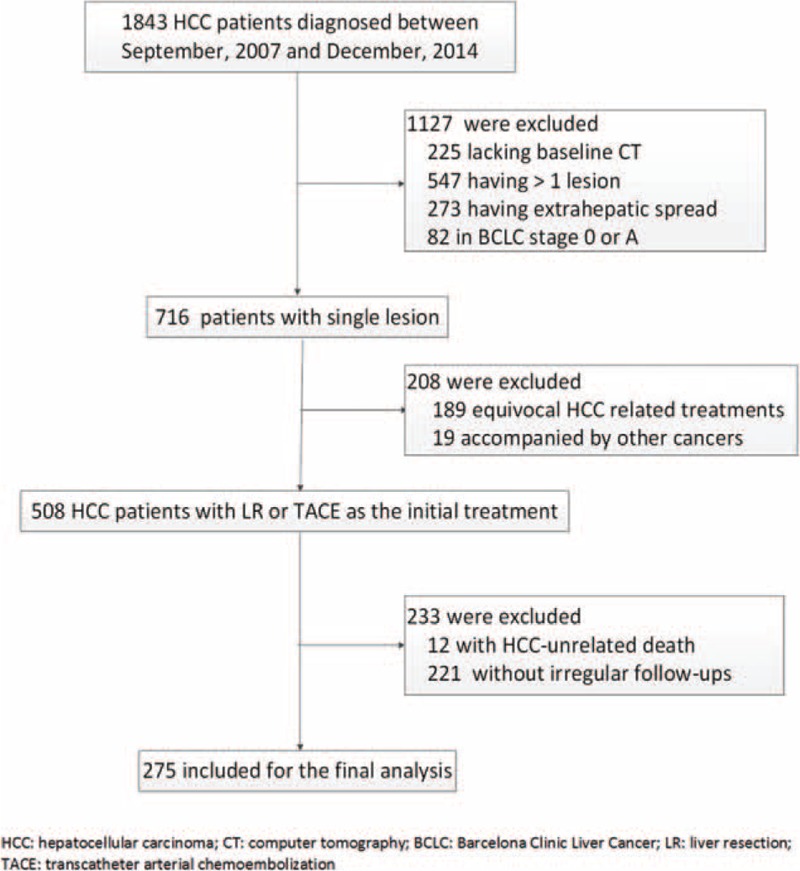
Flowchart illustrating patient inclusion and exclusion criteria. BCLC = Barcelona Clinic Liver Cancer staging system, CT = computed tomography, HCC = hepatocellular carcinoma, LR = liver resection, TACE = transcatheter arterial chemoembolization.

The inclusion criteria were: HCC diagnosed clinically or on pathology; BCLC B or C stage, initially treated with LR or TACE; at least 3 months expected postoperative survival; a follow-up period >3 years if still alive by the cut-off date (March 2015); be regularly followed before death; and availability of a baseline tri-phase hepatic CT image with 5 mm thickness. The exclusion criteria were: lack of baseline CT scans; HCC with multiple lesions; extrahepatic metastasis at diagnosis; suspected or unknown HCC-related treatment history; and comorbidity with other cancers. A flowchart detailing patient selection is shown in Figure 1.

All baseline CT images were undergone within 14 days before initial treatment. Afterwards, patients were followed every 4 to 8 weeks for routine laboratory tests, standard chest radiography, and abdominal CT. All patients received TACE, ablation, or percutaneous ethanol injection (PEI) as necessary after the initial treatment.

### Outcomes

The primary outcome was overall survival (OS), which was defined as the time interval from diagnosis to death. The secondary outcome was time to progression (TTP), which was defined as the time interval from diagnosis to disease progression (PD). PD was defined radiologically according to modified Response Evaluation Criteria in Solid Tumors guidelines for HCC.^26^ Patients alive or with no PD by the end date of this study were censored.

### CT Examinations

All CT images were extracted from our picture archiving and communications system, and were performed by a single scanner (LightSpeed VCT-64, GE Medical Systems, Waukesha, WI) with a nonionic contrast medium (Iopamiro 370) at 1.5 mL/kg according to weight, with a maximum dose of 100 mL injected at 3.5 mL/s. Per the standard imaging protocol at our institution, the following scanning parameters were used: 120 kV; automatic mA; pitch, 0.984; rotation time, 0.6 s; collimation, 5 mm; field of view, 300; and matrix, 512.

### Corona and Mosaic Features

**FIGURE 2:**
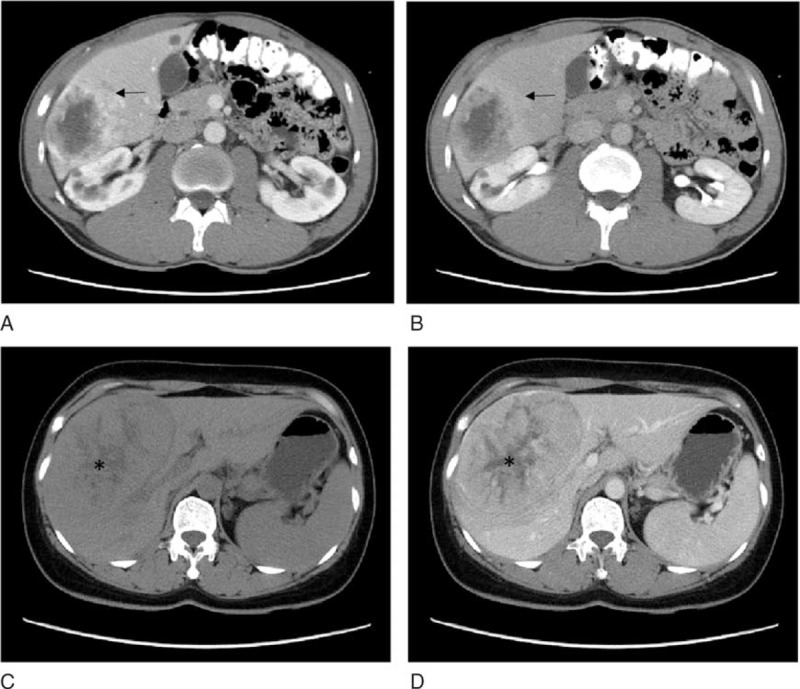
Illustrations of corona enhancement and mosaic architecture. Corona (arrow) manifested as a peritumor enhancing rim at the portal venous phase (A), the transient enhancement fades at delayed phase (B). Computed tomography on a plain scan (C) shows a mosaic architecture (∗) with lower attenuation within tumor. After enhancement, this pattern had slight enhancement and was more clear with low enhancement attenuation relative to tumor at portal venous phase (D).

Typical imaging features of corona (corona− or corona+) and mosaic (mosaic− or mosaic+) patterns were consensus-classified by 2 radiologists with 5 and 4 years of experience in abdominal CT interpretation, respectively (Figure 2). They were both blinded to clinical and pathological information.

### Clinical Data

Candidate variates included age, sex (male or female), cirrhosis (yes or no), BCLC stage, maximum diameter, Child-Pugh class (A or B), alanine aminotransferase (ALT), alpha fetoprotein (AFP) level (<25, 25–400, and >400 μg/mL), corona enhancement (absence or presence), mosaic architecture (absence or presence), subsequent TACE (for OS only; yes or no), ablation (for OS only; yes or no), PEI (for OS only; yes or no), and subgrouping (for the whole population only).

### Statistical Analysis

Statistical analysis was performed by using SPSS 20.0 software (IBM SPSS Statistics, Armonk, NY). Independent-sample *t* tests were used for quantitative data with normality, Mann–Whitney *U* tests were used for ranked data or quantitative data without normality, and chi-squared tests were used for binomial distribution data.

Survival curves were generated using the Kaplan–Meier method and compared by the log-rank test. Afterwards, univariate and multivariate Cox regression was used to identify the independent factors predictive of survival. A 2-sided *P* value < 0.05 was considered statistically significant.

## RESULTS

### Patients

Between September 2007 and December 2014, 1843 patients were diagnosed with HCC by hepatic CT at our institution. After screening, 275 patients were included in this study, of which 181 were treated by LR and 94 by TACE. Twenty-one cases requires a third judge; 10 for corona enhancement (6 in LR, 4 in TACE), 8 for mosaic architecture (5 in LR and 3 in TACE), and 3 for both corona and mosaic patterns (2 in LR and 1 in TACE).

The baseline characteristics of the patients are summarized in Table 1. The majority cause of disease was hepatitis B virus infection, accounting for 74.6% and 79.8% of LR and TACE patients, respectively. Cirrhosis was identified in 66.9% and 75.5% of LR and TACE patients, respectively. There were no significant differences in any of the demographic details or characteristics.

**TABLE 1:**
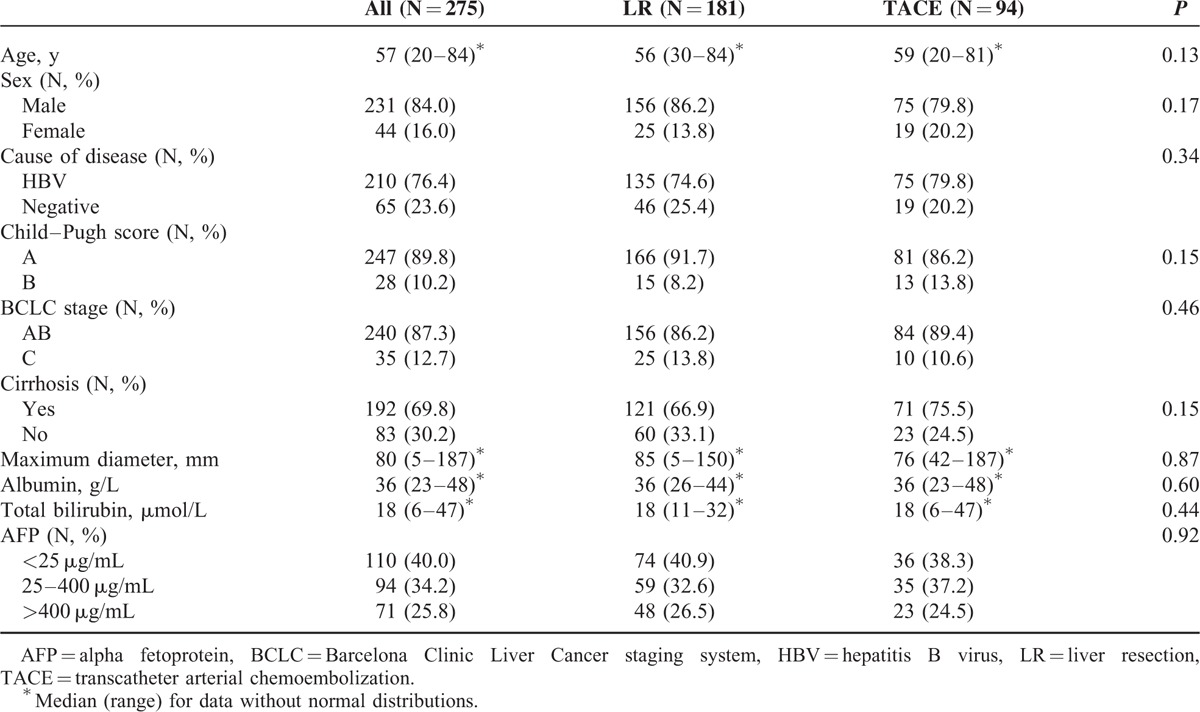
Patient and Tumor Characteristics

For post-therapies, since both our clinical practice and literatures proved that the combination of TACE, ablation, and PEI could bring increased survival benefit,^27–29^ it was a standard protocol to recommend subsequent ablation and PEI as necessary after TACE for patients with large HCCs at our hospital to ensure efficacy. Therefore, more patients received ablation and PEI in the TACE group than in the LR group; 48.6% of LR patients and 100% of TACE patients received subsequent TACE. In the LR group, 17 (9.4%) versus 32 (34.0%) underwent ablation versus PEI, respectively; the rates were 5 (2.7%) versus 17 (18.1%) in the TACE group, respectively.

### Survival Analyses in the TACE Group

**FIGURE 3:**
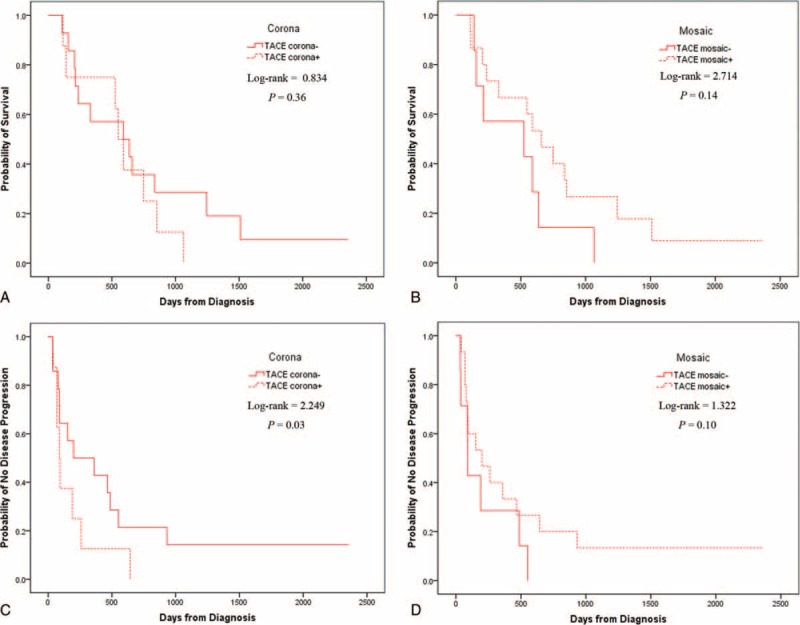
Kaplan–Meier analyses for overall survival (OS) and time to progression (TTP) in the transcatheter arterial chemoembolization (TACE) group. There was no significant difference in OS between TACE corona− and TACE corona+ patients (A), but TTP between these groups was significantly different (C). There was no significant difference between the TACE mosaic− and TACE mosaic+ groups for either OS (B) or TTP (D).

With respect to corona enhancement, 60 patients were TACE corona−, of whom 52 had PD and 51 died; 34 patients were in the TACE corona+ subgroup, all of whom had PD and death. There was no significant difference in OS between the 2 subgroups (*P* = 0.36; Figure 3A, Table 2), but there was a significant difference in TTP (*P* = 0.03; Figure 3C, Table 2). There were no significant differences in demographic characteristics such as BCLC stage, cause or severity of liver disease, Child-Pugh score, or post-therapies between the 2 subgroups.

**TABLE 2:**
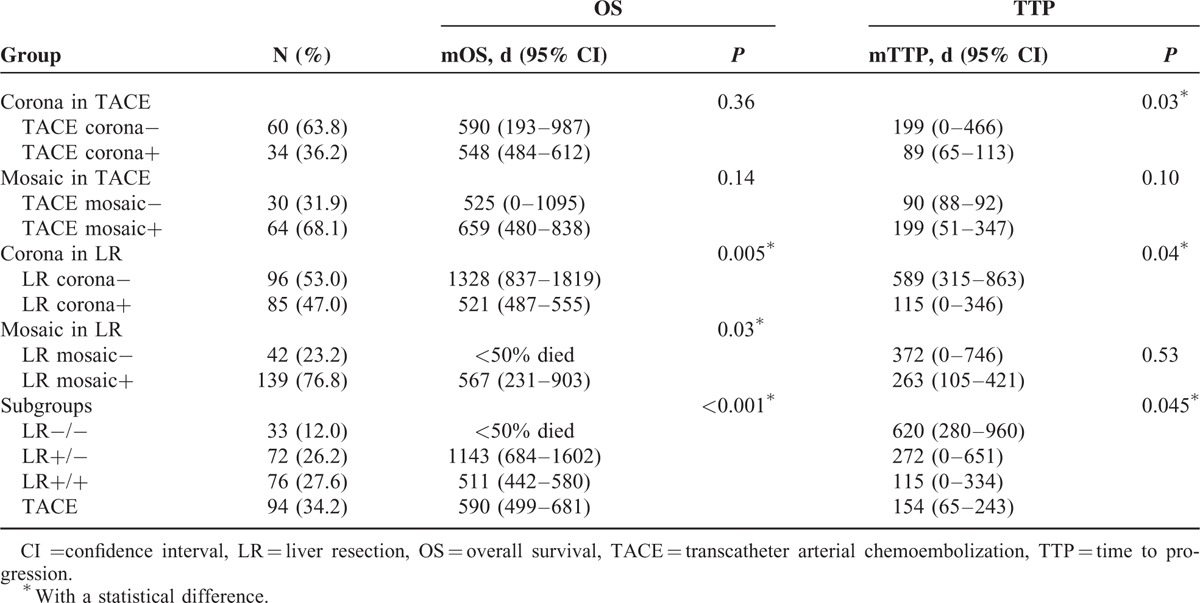
Kaplan-Meier Analysis and Log-Rank Tests

With respect to the mosaic pattern, 30 patients were in the TACE mosaic− subgroup, all of whom had PD and died; and 64 patients in the TACE mosaic+ subgroup, of whom 55 died and 55 had PD. There was no significant difference between the 2 subgroups in OS (*P* = 0.14; Figure 3B, Table 2) or in TTP (*P* = 0.10; Figure 3D, Table 2). Moreover, there were no significant differences in demographic characteristics between the 2 subgroups.

For OS, univariate analysis showed that only sex had a *P* < 0.10; however, there was no significant difference on multivariate Cox regression. For TTP, univariate analysis showed that only ALT had a *P* < 0.10, but there was no significant difference on multivariate Cox regression (Table 3).

**TABLE 3:**
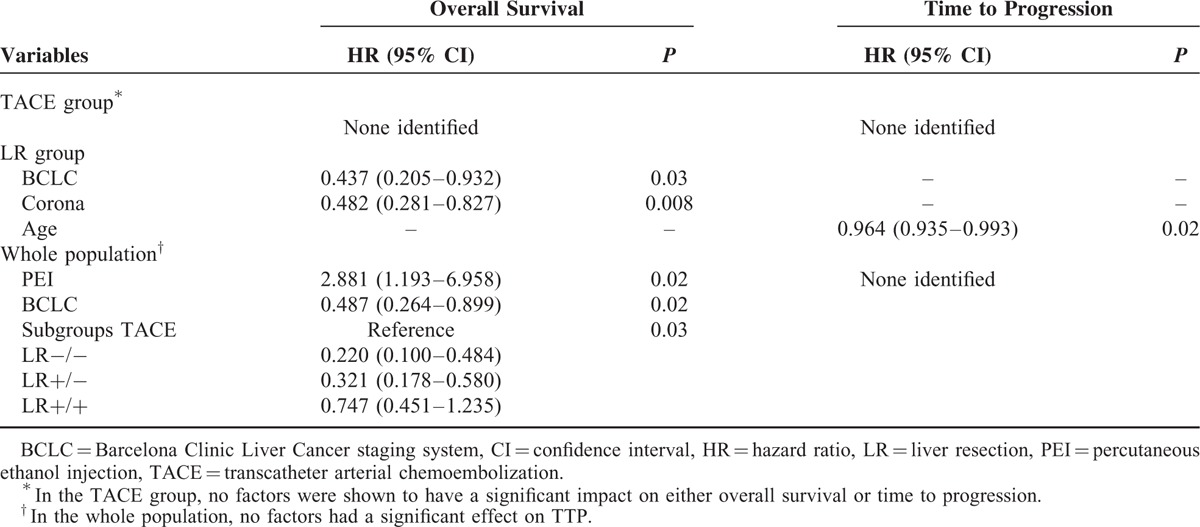
Multivariate Cox Regression in TACE/LR Groups and the Whole Population

### Survival Analyses in the LR Group

**FIGURE 4:**
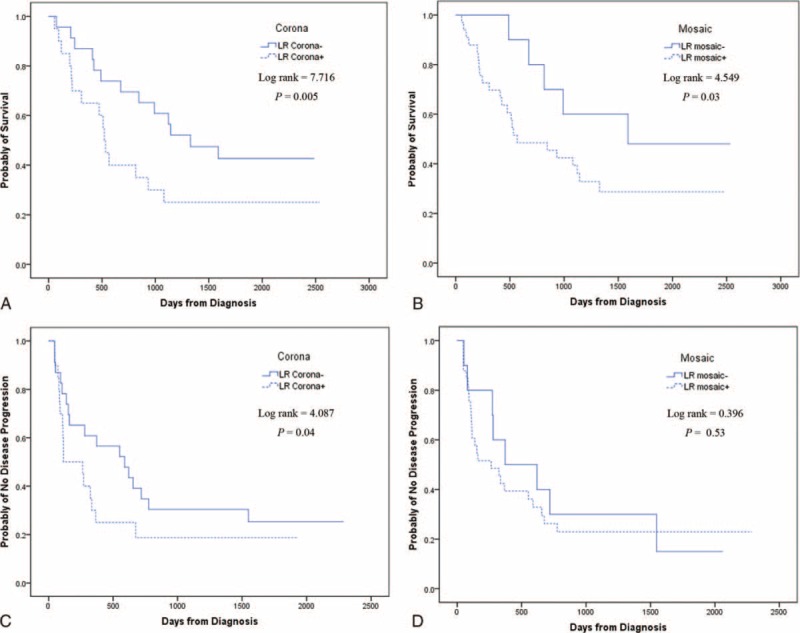
Kaplan–Meier analysis for overall survival (OS) and time to progression (TTP) in the liver resection (LR) group. Both OS (A) and TTP (C) in LR corona− and LR corona+ patients were significantly different. OS between LR mosaic− and LR mosaic+ patients was significantly different (B), but TTP was not (D).

With respect to corona enhancement, the LR corona− subgroup comprised of 96 patients, of whom 54 died and 71 had PD; the LR corona+ subgroup comprised of 85 patients, of whom 64 died and 68 had PD. There was a significant difference in OS between the 2 subgroups (*P* = 0.005; Figure 4A, Table 2), as well as in TTP (*P* = 0.04; Figure 4C, Table 2). There were no significant differences in demographic characteristics between the 2 subgroups.

In terms of mosaic architecture, the LR mosaic− subgroup comprised of 42 patients, of whom 20 died and 34 had PD; the LR mosaic+ subgroup comprised of 139 patients, of whom 98 died and 105 had PD. There was a significant difference in OS between the 2 subgroups (*P* = 0.03; Figure 4B, Table 2), but not in TTP (*P* = 0.53; Figure 4D, Table 2). There were no significant differences in demographic characteristics between the 2 subgroups.

For OS, univariate regression showed that BCLC, corona enhancement, mosaic architecture, and ablation had *P* values < 0.10. After multivariate regression, BCLC and corona enhancement remained statistically significant factors. For TTP, univariate regression showed that only age had a *P* < 0.10; this factor remained statistically significant on multivariate regression (Table 3).

### Survival Analyses in the Whole Population

Since corona and mosaic patterns had limited prognostic value in the TACE group but were significant prognostic factors in LR patients, subgrouping by corona and/or mosaic pattern was not performed for the TACE group (which was used as a reference in this analysis), whereas LR patients were subgrouped into LR−/− (negative for both corona and mosaic patterns), LR+/− (either a positive corona or mosaic pattern), and LR+/+ (positive for both corona and mosaic patterns). The LR−/− subgroup comprised of 33 patients, of whom 16 died and 25 had PD; the LR+/− subgroup comprised of 72 patients (63 corona−/mosaic+ and 9 corona+/mosaic−), with 42 deaths and 55 with PD; the LR +/+ subgroup comprised of 76 patients, of whom 60 died and 59 had PD; and the TACE group comprised of 94 patients, of whom 85 died and 85 had PD. There were no significant differences in demographic characteristics such as BCLC stage, cause or severity of liver disease, or Child-Pugh score between the 4 subgroups.

**FIGURE 5:**
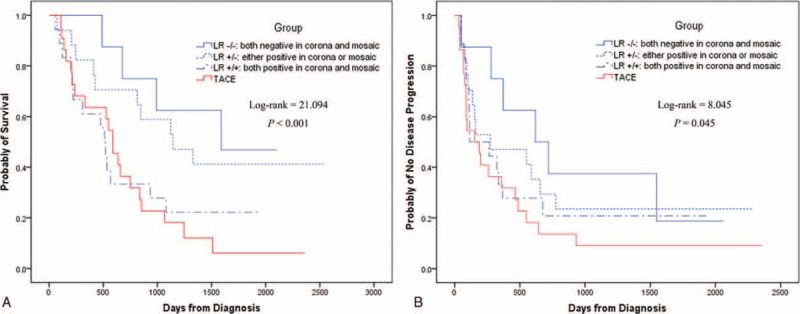
Kaplan–Meier analysis for overall survival (OS) and time to progression (TTP) in the whole population. (A) LR−/− (no corona or mosaic patterns) and LR+/− (1 of either corona or mosaic patterns) had similar OS, which were better than LR+/+ (both corona and mosaic patterns) and transcatheter arterial chemoembolization (TACE) patients; OS was also similar between the latter 2 groups. (B) LR−/− and LR+/− had better TTP than TACE; however, no significant differences in TTP were found between the LR−/− vs LR+/−, LR−/− vs LR+/+, LR+/− vs LR+/+, and LR+/+ vs TACE groups.

The OS between the 4 groups was significantly different (*P* < 0.001). There were significant difference in OS between LR−/− versus LR+/+ (χ^2^ = 7.926, *P* = 0.005), LR−/− versus TACE (χ^2^ = 13.832, *P* < 0.001), LR+/− versus LR +/+ (χ^2^ = 6.338, *P* = 0.012), and LR+/− versus TACE (χ^2^ = 12.734, *P* < 0.001). The OS between the LR−/− versus LR+/− and LR+/+ versus TACE did not show any significant difference (χ^2^ = 0.598, *P* = 0.44; and χ^2^ = 0.029, *P* = 0.87; respectively; Figure 5A, Table 2).

The difference in TTP between the 4 groups was significant (*P* = 0.045). There were also significant differences in TTP between LR−/− versus TACE (χ^2^ = 6.793, *P* = 0.009) and the LR+/− versus TACE (χ^2^ = 3.959, *P* = 0.047). However, no significant differences in TTP were found between LR−/− versus LR+/− (χ^2^ = 0.805, *P* = 0.37), LR−/− versus LR+/+ (χ^2^ = 2.943, *P* = 0.09), LR +/− versus LR+/+ (χ^2^ = 0.505, *P* = 0.48), and LR+/+ versus TACE (χ^2^ = 1.171, *P* = 0.28; Figure 5B, Table 2).

Concerning OS, univariate regression revealed that cirrhosis, BCLC stage, subgroup, TACE, and PEI had *P* values < 0.10. After multivariate regression, PEI, BCLC stage, and subgroup remained significant predictors of OS. No variable had a *P* value < 0.10 for TTP on univariate Cox regression (Table 3).

## DISCUSSION

Although LR is only recommended for HCC patients up to BCLC stage A,^5,30^ 2 recent studies showed that this modality could also offer a survival benefit for patients of more advanced stages.^9,12^ We postulated that the selection of treatment modalities should be made based on results obtained using noninvasive radiological modalities instead of being simply assigned to LR versus TACE. Accordingly, our results showed that both corona and mosaic patterns were related to OS in single-lesion HCC patients of greater than BCLC stage A treated with LR. Furthermore, the combination of the 2 patterns may provide additional information for identifying those patients who would obtain a greater survival benefit from LR.

**FIGURE 6:**
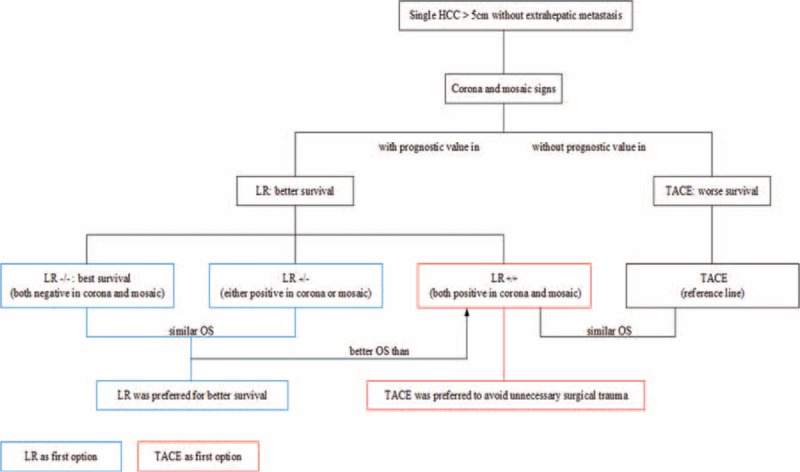
Diagram illustrating the major results and treatment recommendations in this study. HCC = hepatocellular carcinoma, LR = liver resection, OS = overall survival, TACE = transcatheter arterial chemoembolization.

Our results showed that the patients in the LR−/− achieved better prognosis than LR+/+ patients. Moreover, LR−/− patients had a significant survival benefit (for both for OS and TTP), as did LR+/− patients. Meanwhile, LR+/+ had limited survival compared to LR−/− and LR+/−, and showed no significant difference compared to TACE patients. The consequences of these findings may be as follows: for patients negative for both corona and mosaic patterns or positive for only 1 of them, LR should be recommended if possible; and for patients positive for both patterns, LR does not provide a survival benefit compared to TACE. Therefore, considering the possibly of increased perioperative risks and prolonged postoperative recovery, TACE might be recommended as the first-line therapy for patients positive for both patterns (Figure 6).

In our study, both corona enhancement and mosaic architecture were correlated with OS and/or TTP of LR patients on Kaplan–Meier survival analyses. In multivariate Cox regression analysis, however, only corona enhancement was a significant factor for OS. These findings suggested that, although both features conveyed prognostic information for HCC, corona enhancement may be relatively more informative. When examining their roles in patient selection, and considering the 2 studies mentioned above^9,12^ that strongly supported the benefit of LR for HCC patients across various BCLC stages, we believe that the treatment selection process should be aimed to identify patients most likely to benefit from LR; therefore, the LR+/− subgroup was created.

Corona and mosaic patterns are 2 radiological features which could assist with the diagnosis of HCC^18,31^; however, there were limited studies on their prognostic value, as well as their application in treatment selection. Corona enhancement was initially described as a radiological feature differentiating hypervascular hepatic pseudolesions from HCC.^32^ Since then, its value in HCC diagnosis has gradually become appreciated.^18,31^ Pathologically, mosaic architecture was recognized as a pattern reflecting viable tumor nodules and necrotic changes^33–35^ that also had some prognostic value in HCC. Moreover, studies showed that tumor angiogenesis status^36,37^ and heterogeneity^38,39^ might affect the survival of HCC patients. Thus, there are good reasons to believe that corona and mosaic patterns might convey prognostic information and play a role in patient selection for HCC treatment.

Interestingly, corona enhancement can be confused with a peritumor capsule in its appearance; the distinction between the 2 patterns is that the corona pattern fades in the hepatic venous phase while a peritumor capsule manifests as a progressively enhancing rim at delayed phase. Similarly, mosaic architecture may tend to be confused with necrosis, which manifested as hyperintensity on T2 weighed images. Proper recognition of these 2 patterns is crucial in image interpretation. In our study, a third radiologist was consulted to obtain consensus evaluation and reduce the probability of misinterpretation.

As mentioned before, corona enhancement is related to portal venous drainage around the tumor. We postulated that apparently normal tissue around a tumor may actually contain microvascular satellite tumors. Thus, corona enhancement may cause the underestimation of a sufficient LR margin, and therefore hamper complete resection. On the other hand, mosaic patterns related to fibrotic separations within a tumor can be simultaneously removed during LR. As a result, the existence of corona enhancement might be a more reliable prognostic indicator than mosaic architecture in LR patients. Meanwhile, compared to the curative treatment of LR, TACE was more palliative and often had remnant viable HCC. Thus, the amount of remnant HCC may constitute a more important prognostic factor than corona and mosaic patterns. Neither corona enhancement nor mosaic architecture was of prognostic value in the TACE group.

In the whole population, LR−/− patients would be expected to have better OS than LR+/−; however, in our study, the 2 groups had similar survival rates. This may be because, while the LR+/− subgroup included corona−/mosaic+ and corona+/mosaic− patients, 87.5% of the patients in the whole population were corona−/mosaic+. Considering this imbalance, the influence of corona enhancement might be disproportionate in LR corona−/mosaic+ patients, causing their survival outcomes to be similar to the LR−/− subgroup. Owing to the limited sample size of corona+/mosaic− (9 patients; 12.5% of the entire LR+/− subgroup), further studies separating the LR+/− subgroup into corona−/mosaic+ versus corona+/mosaic− may provide a more detailed conclusion.

### Limitations

Our study has some limitations. First, being a secondary project to our previously published study, we only included HCC patients with a single lesion. This limited the sample size on one hand, and resulted in our conclusions vulnerable for patients with multiple lesions on the other. Second, both corona and mosaic patterns were imaging features identified subjectively; hence, radiologist experience was crucial for accuracy. Still, it was noteworthy that, although histological biomarkers may be more objective, they could only be obtained by biopsy, which would have increased the risk of intratumoral bleeding. Noninvasive radiologic markers had the advantage of conventional follow-up without additional burdens on patients. Third, selecting LR versus TACE for tumors with BCLC stages higher than A is a controversial issue. Therefore, a retrospective study from a single center cannot provide a definitive conclusion, and multicenter prospective studies are warranted.

## CONCLUSIONS

Although further validation is warranted before broad clinical adoption, our study demonstrated that the presence of corona and mosaic patterns may indicate limited efficacy of LR for patients with single HCC tumors more advanced than BCLC stage A. Moreover, when patients had both corona and mosaic features, treatment with TACE instead of LR had no negative influence on survival.
